# Interaction of the Oncofetal Thomsen–Friedenreich Antigen with Galectins in Cancer Progression and Metastasis

**DOI:** 10.3389/fonc.2016.00079

**Published:** 2016-03-31

**Authors:** Paulina Sindrewicz, Lu-Yun Lian, Lu-Gang Yu

**Affiliations:** ^1^Gastroenterology Unit, Department of Cellular and Molecular Physiology, Institute of Translational Medicine, University of Liverpool, Liverpool, UK; ^2^NMR Centre for Structural Biology, Institute of Integrative Biology, University of Liverpool, Liverpool, UK

**Keywords:** galectin, TF antigen, metastasis

## Abstract

Aberrant glycosylation of cell membrane proteins is a universal feature of cancer cells. One of the most common glycosylation changes in epithelial cancer is the increased occurrence of the oncofetal Thomsen–Friedenreich disaccharide Galβ1–3GalNAc (T or TF antigen), which appears in about 90% of cancers but is rarely seen in normal epithelium. Over the past few years, increasing evidence has revealed that the increased appearance of TF antigen on cancer cell surface plays an active role in promoting cancer progression and metastasis by interaction with the β-galactoside-binding proteins, galectins, which themselves are also frequently overexpressed in cancer and pre-cancerous conditions. This review summarizes the current understanding of the molecular mechanism of the increased TF occurrence in cancer, the structural nature, and biological impact of TF interaction with galectins, in particular galectin-1 and -3, on cancer progression and metastasis.

## Introduction

Glycosylation is one of the most common post-translational modifications of cell membrane proteins (1). Among the two major types of protein glycosylation in human cells, the N-linked glycosylation is characterized by attachment of carbohydrates to the amide group of asparagine residues of proteins in the consensus sequence Asn–X–Ser/Thr, whereas in the classical *O*-linked mucin-type glycosylation, *N*-acetyl-galactosamine is covalently linked to the hydroxyl group of serine or threonine residue of the protein backbone (2, 3). *O*-linked protein glycosylation occurs during protein movement through ER–Golgi pathway by sequential incorporation of monosaccharides; this process is controlled by various factors, such as relative abundances of glycosyltransferases and glycoprotein substrates, as well as the availability of sugar-donor molecule (2, 3). Altered glycosylation of cell membrane proteins is a hallmark of cancer and a universal feature of oncogenesis and cancer progression (2, 4). Such glycosylation changes typically include the occurrence of incomplete or truncated glycan structures, accumulation of glycan precursors, or the presence of novel tumor-specific carbohydrate epitopes. More and more evidence shows that these glycosylation changes on cancer cell surface are critically involved in the regulation of cell social behaviors and is increasingly recognized to play an important role in cancer development, progression, and metastasis (2). One of the most prevalent glycosylation alterations in human carcinomas is the increased occurrence of short carbohydrate structure Galβ1–3GalNAcα, also known as the oncofetal Thomsen–Friedenreich (TF or T) antigen. The TF antigen is the core I structure of *O*-linked mucin-type glycans. In normal epithelium, TF structure is masked by other sugar residues to form branched and complex glycans or being modified by sialidation, sulfation, or fucosylation (5, 6). Low level of TF expression was occasionally reported in normal epithelium (7), but this was suspected to be related to the use of less/different specific analytic tools in the studies. Peanut agglutinin, one of the most commonly used analytic tools to detect TF expression in tissue sections, is known to also recognize other sugar structures (although with low affinity) such as terminal galactose and Galβ1 → 4GlcNAc (8). Different anti-TF antibodies also show to bind TF-related structures differently (9). Un-masked and unsubstituted TF structure is known to occur in about 90% of all cancers (5). There is a considerable amount of literature showing a positive correlation between the occurrence of TF and tumor progression in various types of cancer (10–12).

## Molecular Mechanisms Leading to Increased TF Occurrence in Cancer

Despite unequivocal evidence of the widespread appearance of TF in cancer cells, the exact mechanisms underlying this increased TF availability in cancer is not fully understood. Biosynthesis of the mucin-type *O*-linked glycans occurs by transferation of individual carbohydrate moieties from nucleotide sugar donor molecules to the acceptor in a stepwise manner in the Golgi apparatus (6, 13). TF antigen is an intermediate structure in the biosynthesis of complex *O*-linked oligosaccharides. Its formation is carried out by the addition of galactose (Gal) from UDP-Gal to the precursor structure GalNAc (Tn antigen) by core 1 β1,3-galactosyltransferase, also known as T synthase (13, 14). It is believed that unbalanced expression of glycosyltransferases involved in the glycosylation pathways, such as T synthase, β1,6-GlcNAc-transferase, sulfotransferase, and sialyltransferases, as well as altered availability of precursor monosaccharide ­molecules, is responsible for altered glycosylation patterns in cancer and thus are probably the main factors responsible for enhanced availability of TF disaccharide (15–19). In addition, changes in acidification of Golgi apparatus have been shown to correlate to enhanced TF expression in breast and colorectal cancer cells (20). Furthermore, the availability and activity of molecular chaperone Cosmc, which is required for proper folding of a functional T synthase (21), also influences the overall TF appearance in cancer. Mutations of Cosmc in human colon carcinoma LSC cells have been shown to account for the reduced T synthase activity and enhanced occurrence of Tn and sialyl-Tn antigens (22). Cosmc knockdown in pancreatic cells was seen to lead to aberrant O-glycosylation and acquisition of oncogenic properties (23). Forced expression of Cosmc in HCT116 colon cancer cells resulted in enhanced TF expression and increased cell growth, migration, and invasion (24). It should be noted, however, that in breast cancer cells, TF antigen was seen to be still expressed along with Tn and sialyl-Tn antigens in the absence of Cosmc, suggesting that the chaperone function of Cosmc is not a sole determinant in the enhanced expression of TF in those cells (14).

## TF Antigen-Expressing Proteins in Cancer

A number of proteins are known to carry the unsubstituted TF structure in cancer. These include the adhesion molecules CD34 and CD44 (25, 26) and the transmembrane mucin proteins MUC1 and MUC4 (5, 27, 28). Interestingly, CD34 and CD44 are also known as cancer stem cell markers, which are unique for specific types of cancer, for example, CD34 in leukemia and sarcoma, whereas CD44 in colon and breast cancer (5, 29). CD34, identified as hematopoietic cell surface antigen, is a transmembrane protein involved in cell adhesion and homing of leukocytes to the endothelium during inflammatory responses (30), whereas CD44 is a major hyaluronan receptor that is involved in hematopoietic stem cell and leukemia-initiating cell homing and migration (31). The TF-bearing protein MUC1 is a large and heavily glycosylated transmembrane mucin protein that is overexpressed by most cancer cells of epithelial origin (2, 32). Cancer-associated MUC1 also carries other truncated oligosaccharides such as Tn and sialyl-Tn antigens (33). In contrast to its polarized localization on the apical surface of epithelial cells in normal epithelium, cancer-associated MUC1 loses its apical localization and is expressed on the entire cell surface (2, 34).

Over the past decade, more and more evidence has revealed that the pan-carcinoma-associated TF antigen is a natural ligand of the galactoside-binding galectins and the TF–galectin interaction influences a number of key steps in cancer progression and metastasis.

## Galectins

Galectins are a family of 15 (so far) mammalian β-galactoside-binding proteins. Each galectin member contains one or two well conserved carbohydrate recognition domains (CRDs) that recognize galactose-terminated glycans (35, 36). Galectins are widely expressed by human cells and are divided into three subgroups of proto-type, tandem repeat type, and chimera type based on their structural differences. The prototype galectins include galectin-1, -2, -5, -7, -10, -11, -13, -14, and -15, each with one CRD. The tandem repeat type galectins include galectin-4, -6, -8, -9, and -12, each contains two CRD connected by a short linker region. Galectin-3, the only chimera type galectin, consists of one CRD at its C-terminus and an extended and flexible N-terminal (35, 37). The N-terminal domain of galectin-3, which is often referred to as collagen-like domain, is responsible for galectin-3 ­multimerization upon galectin-3 contact with multivalent ligands (38, 39). Despite sharing conserved CRDs, galectins often display significant differences in their binding specificities to branched, repeated, or modified galactose residue (40, 41). These binding differences of galectin members toward oligosaccharides are believed to have implications in their biological activities in physiological and pathological conditions (41).

A number of galectins show altered expression, very often increased expression, in tumor cells compared with their normal counterparts. In many cases, the altered expression of galectins correlates to the acquisition of aggressive and metastatic phenotype (42, 43). There is a large body of evidence showing that galectins play important roles in tumor transformation, cancer cell adhesion, invasion, migration, and angiogenesis through multiple mechanisms (44). These divergent actions of galectins are partly derived from the ability of galectins to interact with various galactose-terminated glycans in different environments.

## Interaction of Cancer-Associated TF with Cancer- and Endothelial-Associated Galectins in Cancer Progression and Metastasis

In 2000, Glinsky and colleagues first reported an interaction of TF with galectin-1 and -3 in cell–cell homotypic aggregation and cell adhesion to cultured human vascular endothelial cells of human breast and prostate cancer cells. They showed that these galectin-mediated cell–cell interactions were inhibited by the presence of TF-binding peptide P-30, TF-antigen mimicking compound lactulosyl-l-leucine, or anti-TF monoclonal antibody (45–47). Interaction of cancer-associated TF with endothelium-associated galectin-3 showed to affect initial stages of cancer cell–endothelium adhesion (45–47). Several other investigations subsequently also reported a role of galectin-3–TF interaction in mediating cancer cell adhesion to the endothelium *in vitro* and in mice (48–50). Inhibition of the galectin–TF interaction by anti-TF antibody, anti-galectin-3 antibody, modified citrus pectin, or lactulosyl-l-leucine suppressed the adhesion of human breast carcinoma cells to HUVECs and endothelial bone marrow cells 60 (HBMECs-60) *in vitro* and in *ex vivo* perfused porcine dura mater model (48).

Cancer cell–endothelial interaction mediated by cancer cell-associated TF was also shown to enhance expression of endothelial cell surface-associated galectin-3, resulting in increased adhesion of breast and prostate cancer cells to the endothelium of intact well-differentiated micro-vessels (51). The same phenomenon of galectin-3 cell surface mobilization in endothelial cells was noticed in cell response to the highly metastatic, TF antigen-expressing MDA-MB-435 cells but not in TF antigen-deficient MDA-MB-468 cells (45). These discoveries suggest that cancer cell-associated TF serves not only as a ligand for endothelia-associated galectin-3 in cell adhesion but also as an activator for endothelial translocation of intracellular galectin-3 to the cell surface, where it may prime the endothelium for subsequent binding/docking of circulating tumor cells in metastasis (45).

## Interaction of Cancer-Associated TF Antigen with Circulating Galectins in Metastasis

Over expression of MUC1 as well as increased occurrence of TF antigen carried by MUC1 are both characteristic features of epithelial cancer cells. Increased levels of circulating galectin members, in particular galectin-3, are also commonly seen in cancer patients (52). Patients with metastasis are seen to have higher galectin-3 serum concentration than those with localized tumors (52). Studies over the past 10 years have shown that the increased levels of galectins in the bloodstream, in particular galectin-3, may play an important role in promoting circulating tumor cells hematogenous dissemination to remote tumor sites as a result of their increased interactions with TF presented by MUC1 on the surface of tumor cells. Interaction of galectin-3 with TF on cancer-associated MUC1 causes MUC1 cell surface polarization, resulting in exposure of the underlying smaller adhesion molecules, which are otherwise masked by the large size of MUC1 (53). This showed to lead to increased adhesion of disseminating tumor cells to the blood vascular endothelium (54). Changes of MUC1 cell surface localization in response to galectin-3 binding also induce cancer cell homotypic aggregation and the formation of circulating tumor emboli, thus preventing the cells from undergoing anoikis and prolonging the cell survival (55). It was found that a tiny 3% tumor cell clusters in the total circulating tumor cells could account for strikingly over 50% of the metastasis in a mouse metastasis model, and that the continued presence of even 5% tumor cell clusters in the blood was correlated significantly to reduced overall survival in breast and prostate cancer patients (56). Interaction of galectin-3 with the TF on the mucin protein MUC4 was also reported to produce a similarly enhanced cell adhesion of pancreatic cancer cells (27). Although with different binding affinities, other galectin members also recognize TF antigen and several of them, such as galectin-1, galectin-2, -4, and -8, are shown to also have elevated levels in the circulation of cancer patients (57). It is possible that, like galectin-3, the increased interactions of these galectin members with cancer-associated TF/MUC1/4 in circulation may also influence tumor cell metastatic spread. In support of this, exogenous introduction of recombinant galectin-2, -4, or -8 at broadly pathological concentrations observed in cancer showed to induce changes of MUC1 cell surface localization and increase of cancer cell adhesion to endothelial monolayers in cell culture (57). The reported actions of TF–galectin interaction on cancer cell behaviors in cancer progression and metastasis are summarized in Table 1.

**Table 1:** **Influences of TF–galectin interaction on cancer cell behaviors in cancer progression and metastasis**.

	Consequences of TF–galectin interaction	Reference
Galectin-1	Increased cancer cell–cell homotypic aggregation	(47)
	Increased cancer cell–endothelial adhesion	(47)
Galectin-2	Increased cancer cell–endothelial adhesion	(57)
Galectin-3	Changes of MUC1 cell surface polarization	(54)
	Increased cell–cell homotypic aggregation	(46, 47, 55)
	Increased cancer cell–endothelial adhesion	(27, 45–47, 53)
	Formation of circulating tumor emboli	(55)
Galectin-4	Increased cancer cell–endothelial adhesion	(57)
Galectin-8	Increased cancer cell–endothelial adhesion	(57)

The influence of TF–galectin interaction on cancer progression and metastasis raises the possibility that therapeutic inhibition of such interactions may be a viable strategy to reduce cancer ­progression and metastasis. Several possibilities are currently being explored by a few laboratories. These include the use of anti-TF antibodies, TF-mimicking peptides (49), negative galectin-3 mutants (58), and synthetic (59) and semi-synthetic oligosaccharides (60), and several approaches have shown promise in animal models and are currently undergoing clinical trials.

## The Structural Nature of TF–galectin Interaction

All galectins contain at least one CRD, which is responsible for their binding to galactose-terminated glycans. This conserved sequence, consisting of approximately 130 amino acid residues, adopts a typical β-sandwich motif formed by six-stranded and five-stranded antiparallel β-sheets. The carbohydrate-binding site of galectins is localized within β-strands 1–6 of the concave surface (35, 61–63). Although all galectins share this CRD with well-conserved three-dimensional structure, the quaternary structures of galectins differ and are believed to influence their biological activities (35, 64). Studies using fungal galectin CGL2 from *Coprinopsis cinerea*, mouse galectin-9 N-terminal CRD, and mushroom *Agrocybe aegerita* galectin AAL provided structural insight into the recognition specificity and binding mechanisms between TF disaccharide and galectins (37, 65, 66). Not surprisingly, the galactose moiety of TF disaccharide was found to orientate and interact in the same manner as lactose/*N*-acetyl-lactosamine-derived structures. The GalNAc moiety of the TF antigen is involved in TF interactions with fungal galectin CGL2 and mouse galectin-9 *via* formation of hydrogen bonds with Arg and Glu residues within their binding sites (37, 65). In contrast, studies using mushroom *A. aegerita* galectin AAL revealed specific Arg–water–Glu–water structural motif-based hydrogen bonding network that was unique for galectin–TF antigen association (66).

**Figure 1:**
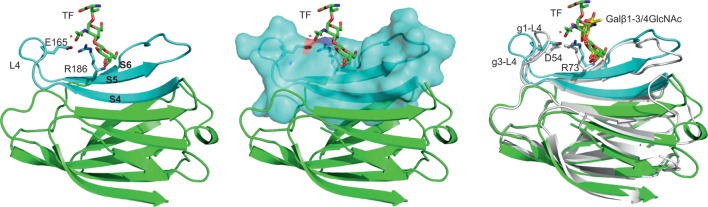
**Structural feature of galectin-3–TF interaction**. Left panel: overall structure of galectin-3 CRD in complex with TF disaccharide (PDB 3AYA) (63). TF disaccharide (in stick model) binds to the CRD concave surf forms a E^165^-water-R^186^-water motif for TF recognition. Residues from strands S4–S6 (colored cyan) interact with the TF; L4 is the loop between S4 and S5. Middle panel: surface representation of strands S4–S6. Left panel: comparison between the structures of galectin-3 complex with the TF disaccharide and galectin-1 complex with Galβ1–3/4GlcNAc disaccharide (PDB 4XB1) (76). Galectin-3/TF is in green; galectin-1/Galβ1–3/4GlcNAc in white cartoon/yellow sticks. g1-L4 and g3-L4 are, respectively, the L4 loop for galectin-1 and galectin-3, which are found to adopt different conformations due to differences in the lengths and amino acid sequences of the two loops. D54 and R73 make up D^54^-water-R^73^-water motif that mediates ligand interactions.

Among human galectins, galectin-1 and -3 gained most attention due to their cancer-related activities. The nature of TF interaction with human galectin-1 and -3 were first reported by Bian and co-workers (63). Their studies revealed differences in binding of the two galectins to TF disaccharides that were attributed to subtle difference in their CRD sequences. Isothermal titration calorimetry analysis showed that the galectin-3–TF interaction (Kd = 47 μM) is two orders of magnitude stronger than galectin-1-TF interaction (Kd = 4 mM). The galectin-3 residues engaged in its interaction with TF occur on strands 4–6 of the concave β-sheet and the loop linking strands 4 and 5 (Figure 1). Currently, there is no experimentally determined galectin-1 complex with TF disaccharide; a recent study has reported galetin-1 in complex with Gal β1-3/4GlNAc (67). Interestingly, detailed analysis of galectin-3 interaction with TF, particularly with the GalNAc moiety, brought to light the importance of hydrogen bond network, previously observed in mushroom galectin AAL. This Arg186-water1-Glu165-water2 structural motif-based hydrogen bond network was suggested to play a role as a major determinant for specific TF recognition and high affinity binding of galectin-3. Importantly, this unique recognition mode was also found in galectin-3 complexes with two TF antigen derivatives, TFN and GM1. The most restricting factor for TF binding to galectin-1 was found to be a pentad residue motif ^51^AHGDA^55^ at the loop 4 linking β-strands 4 and 5, which was shown to force the loop to adopt different conformation from loop 4 in galectin-3. In addition, the His residue within the galectin-1 sequence exerts a serious steric hindrance for the carbohydrate binding. Mutagenesis experiments confirmed the pentad residue sequence within galectin-1 loop 4 to be the major factor influencing the difference between bindings of galectin-1 and galectin-3 to TF structure (63).

The TF disaccharide is linked to its carrier proteins *via* serine or threonine residues in glycans. Galectin interaction with TF has been reported to include galectin interaction with the TF-protein backbone, which additionally enhances the binding (68–70). Structural analysis on binding of avian galectin-3 to TF-threonine conjugate shows transient interaction between galectin-3 and amino acid threonine (71). Kinetic analysis reveals that the linkage of TF disaccharide to MUC1 fragments influenced the thermodynamic binding profile of galectin-3. The binding affinity of galectin-3 to TF-carrying glycans showed to be more than fivefold higher than to free TF disaccharide (72). These discoveries are in line with previous observations of other carbohydrate-binding proteins showing that lectin binding to glycoconjugates tends to be stronger than binding to carbohydrates (73). It should be stressed that all the information obtained so far for the TF–galectin-3 interaction were conducted using the CRD domain of galectin-3 and no information is yet available for full-length galectin-3. As the protein backbone of TF-expressing proteins is actively involved in galectin binding to TF-expressing glycans, future studies with full-length galectin-3 will be paramount to understand the exact mechanisms of the TF–galectin-3 interaction at molecular and submolecular levels.

Another feature of the galectin–TF interaction is protein multimerization. Cross-linking of multivalent glycoconjugates and receptors can lead to increase in galectin binding affinity. This phenomenon is known to play an important role in providing the required affinity and specificity of galectin actions (74). Isothermal titration micro-calorimetry analysis of galectin binding to multivalent carbohydrates by Dam et al. suggested that galectin-induced glycan clustering could enhance subsequent binding events by as much as 10,000-fold (75). So, even though galectin binding to certain glycans shows low affinity *in vitro*, their interactions in physiological and pathological conditions may be much stronger and hence functionally significant.

## Conclusion Remarks

The occurrence of the oncofetal TF antigen and the increased expression of galectins are both common features in cancer. More and more evidence has revealed that TF antigen is a natural ligand of galectins in cancer, and the galectin–TF interaction promotes a number of key steps (e.g., cancer cell heterotypic adhesion and homotypic aggregation) in the cancer progression and metastasis. Such a cancer-associated molecular interaction, as a result of highly specific occurrence of TF antigen in cancer cells, offers a potential therapeutic target for the development of novel strategies for cancer treatment. Moreover, as galectins are widely expressed by many types of human cells, cancer-associated TF may interact with galectins expressed by other cell types (e.g., immune cells) and influence cancer progression and metastasis. Further investigations in these areas are warranted. Structurally, more studies are needed to understand the galectin–TF interaction at atomic levels using full-length galectins.

## Author Contributions

All authors contributed to the preparation of the manuscript, and all read and approved the final version of the manuscript.

## Conflict of Interest Statement

The authors declare that the research was conducted in the absence of any commercial or financial relationships that could be construed as a potential conflict of interest.
